# ProTect: a hybrid deep learning model for proactive detection of cyberbullying on social media

**DOI:** 10.3389/frai.2024.1269366

**Published:** 2024-03-06

**Authors:** T. Nitya Harshitha, M. Prabu, E. Suganya, S. Sountharrajan, Durga Prasad Bavirisetti, Navya Gadde, Lakshmi Sahithi Uppu

**Affiliations:** ^1^Department of Computer Science and Engineering, Amrita School of Computing, Amrita Vishwa Vidyapeetham, Chennai, India; ^2^Department of Information Technology, Sri Sivasubramaniya Nadar College of Engineering, Chennai, India; ^3^Department of Computer Science, Norwegian University of Science and Technology (NTNU), Trondheim, Norway

**Keywords:** cyber bullying, deep learning, social media, text analysis, neural network, machine learning, data mining

## Abstract

The emergence of social media has given rise to a variety of networking and communication opportunities, as well as the well-known issue of cyberbullying, which is continuously on the rise in the current world. Researchers have been actively addressing cyberbullying for a long time by applying machine learning and deep learning techniques. However, although these algorithms have performed well on artificial datasets, they do not provide similar results when applied to real-time datasets with high levels of noise and imbalance. Consequently, finding generic algorithms that can work on dynamic data available across several platforms is critical. This study used a unique hybrid random forest-based CNN model for text classification, combining the strengths of both approaches. Real-time datasets from Twitter and Instagram were collected and annotated to demonstrate the effectiveness of the proposed technique. The performance of various ML and DL algorithms was compared, and the RF-based CNN model outperformed them in accuracy and execution speed. This is particularly important for timely detection of bullying episodes and providing assistance to victims. The model achieved an accuracy of 96% and delivered results 3.4 seconds faster than standard CNN models.

## 1 Introduction

When social media initially emerged, it was seen to be a blessing. They connected individuals from all over the world and made communication more reliable and rapid. Cyberbullying is among the most alarming problems that have arisen due to the popularity and effectiveness of these platforms. Cyberbullying is a form of harassment or abuse directed toward regular users of these networks by those who exhibit psychotic symptoms. Blackmail, verbal abuse, and hate speech are just a few ways harassment can manifest. Bullying has been a widespread problem in society for a long time, but the increased rates of bullying and cybercrime point to a wide range of opportunities for abusers to conceal their identities behind virtuality (Raj et al., 2021). Bullying frequently undermines communal integrity and results in severe psychological problems, including hatred, wrath, and sadness, with extreme examples of harassment even resulting in homicide. Cyberbullying victims typically depict online reporting mechanisms as ineffective because they frequently fail to comprehend and identify their problems.

Over the last decade, cyberbullying crimes have inflated from 20.8% (performed on a sample size of 4,441) to 45.5% (performed on a sample size of 2,546) according to Patchin's information gathered and reported online in 2022. With a decrease in the number of subjects for the research, this inverse trend clearly shows a trend toward increased instances of cyberbullying. More than 30% of the casualties were schoolchildren and other people under 18. These youngsters typically fall into such notorious online activities due to a lack of awareness and require stringent measures to assess their safety on social platforms (Samghabadi, 2020). Therefore, it becomes necessary to employ a specific automated approach that can detect such egregious occurrences without needing to rely on or submit to human supervision. With such small datasets, these models appear to function quite well. However, when they were used with real-time datasets, such as those used in this study, they could not handle excessively noisy data and achieved a different degree of accuracy (Kumar and Sachdeva, 2022). Hence, the primary objective is to build a classification model that works well on small-scale and real-time datasets while maintaining fair accuracy.

This study primarily focuses on the problem of assessing the impact of contemporary intelligent algorithms on highly complicated and unstructured real-time datasets acquired from leading researchers globally. To demonstrate the generic approach (Kumari and Singh, 2021) of the suggested model, which may assist with text-based classification tasks on other platforms as well, it is applied to two distinct types of datasets from Instagram and Twitter. To confirm the efficacy of the suggested model, this dual testing was formed on further deep learning (DL) and machine learning (ML) models from the literature review. From the results, it can observed that our model outperformed the existing baseline models by 13% in terms of accuracy and was faster in execution.

This article proposed the problem of cyberbullying detection on social media and the challenges of applying machine learning and deep learning techniques to real-time datasets. Also stated main objective of developing a hybrid CNN-RF model that can work on both small and large datasets with high accuracy and speed. In related work, reviewed the existing literature on cyberbullying detection using various ML and DL models, such as random forest, SVM, naïve Bayes, RNN, CNN, and self-attention models. Also presented a graphical analysis of the most frequently used algorithms for text classification.

In proposed framework, described the datasets used for the study, the pre-processing steps involving NLP and vectorization techniques, and the overall workflow of the proposed methodology. Also explained the novel CNN-RF architecture and its algorithmic implementation in detail. With that presented the comparative analysis of the results obtained from the implementation of various algorithms on the selected datasets and used the evaluation metrics such as accuracy, precision, recall, F1-score, and execution time to measure the performance of the models (Kastrati et al., 2019). In conclusion, proved that the proposed CNN-RF model outperforms all the other models in terms of accuracy and speed.

## 2 Related work

Since 2011, researchers have actively investigated ML and DL methodologies toward text classifications to classify social media content as bullying or non-bullying. The initial research phase was based on supervised ML approaches combined with natural language processing (NLP) technologies for character-level representations such as bag-of-words. The work of applying ML algorithms toward cyberbullying detection has been mostly limited to comparing baseline algorithms to prove which works best on presented data. Islam et al. (2020) studied the significance of individual features for efficient Twitter cyberbullying classification. Their study depicts that support vector machines (SVMs) were the best-suited models with the highest accuracy. However, a different set of authors (Keni and Kini, 2020; Kumar, 2020; Gummadavelly et al., 2021) who worked with a similar dataset based on Twitter concluded that random forest and naïve Bayes with poly kernel along with an SVM classifier have superior accuracy toward supervised bullying detection tasks. The first Instagram-based (artificial dataset) study was carried out by Hosseinmardi et al. (2015), wherein naïve Bayes and SVM classifiers were employed, and it was concluded that SVM works best with multimodal data.

With further room for improvement, researchers looked into the deep learning domain for this complexity and effectiveness on large datasets. The authors of Chia et al. (2021) focused on identifying text with irony and sarcasm in a dataset extracted from Twitter. To detect these data, machine learning and feature engineering techniques were used. For an optimized solution, numerous methods were used, starting with the primary classifier Naïve Bayes, k-nearest neighbor, to obtain fewer errors, JRip, SVM, and CNN were used. CNN gained the highest accuracy among the classifiers, followed by SVM and others. The metrics used to determine these percentages were standard accuracy (A), precision (P), recall (R), and balanced F F-score (F1). Similarly, Nirmal et al. (2021) planned to detect cyberbullying across social media using NLP and machine learning approaches.

Yuvaraj et al. (2021) developed a novel automated classification model that identified cyberbullying texts without fitting the data into sizeable dimensional space. The classification model was an enhanced decision tree classifier combined with DNN to resolve the issue of the limit of tree depth in the novel deep decision tree classifier to minimize the problem of overfitting. Although this approach has shown high-level accuracy of cyberbullying detection, the authors were inspired toward better optimization by exploring deep learning models accessing their reliability on real-world high data. Furthermore, using deep neural networks (DNNs, Raj et al., 2022) proposed a model to detect cyberbullying in tweets and other social media posts. DNNs are effective compared to conventional techniques.

Additionally, several self-attention models have emerged with the rise of cyberbullying cases on social media. The article of Pradhan et al. (2020) worked on self-attention models to explore the adaptivity and efficiency of these models. The methodology used was GloVe vector representation. In this representation of words, each word is represented by a one-hot vector. This one-hot vector is fed into the model with optimized hyperparameters that predict the probability of the post being abusive. GloVe outperformed the state-of-art models and deep learning models. Although the model showed noticeable results, the fact that cyberbullying is not constrained only to texts is also a matter of consideration. Gencoglu (2021) intended to detect cyberbullying using a trained model that could employ fairness constraints. A model from deep neural network architectures where transformer networks were used for binary classification of cyberbullying and for the model to work on multiple languages, sentence-DestilBERT, was used.

A gradually more significant number of DL-based hybrid models (Banerjee et al., 2019; Murshed et al., 2022) to identify and classify cyberbullying accurately started to come into existence. Aspects such as the time taken to detect such incidents and scalability to the rate at which content is created on social media are hardly considered. Yao et al. (2019) worked on the issues mentioned above. CONcISE was a model developed that used a sequential hypothesis testing formulation that acutely reduced the number of features for classifying comments while maintaining a high level of classification accuracy, timeliness, and efficiency. Despite the outstanding results of the model, the fact to be noted is that experimental results were only carried out using a single dataset. The model showed impotence toward the validation of labels in the dataset. Extending the approach toward detection timeliness, López-Vizcaíno et al. (2021) investigated a supervised learning method in accordance with threshold and dual. Threshold and dual detection models that detect cyberbullying more quickly involve two feature groups.

**Figure 1:**
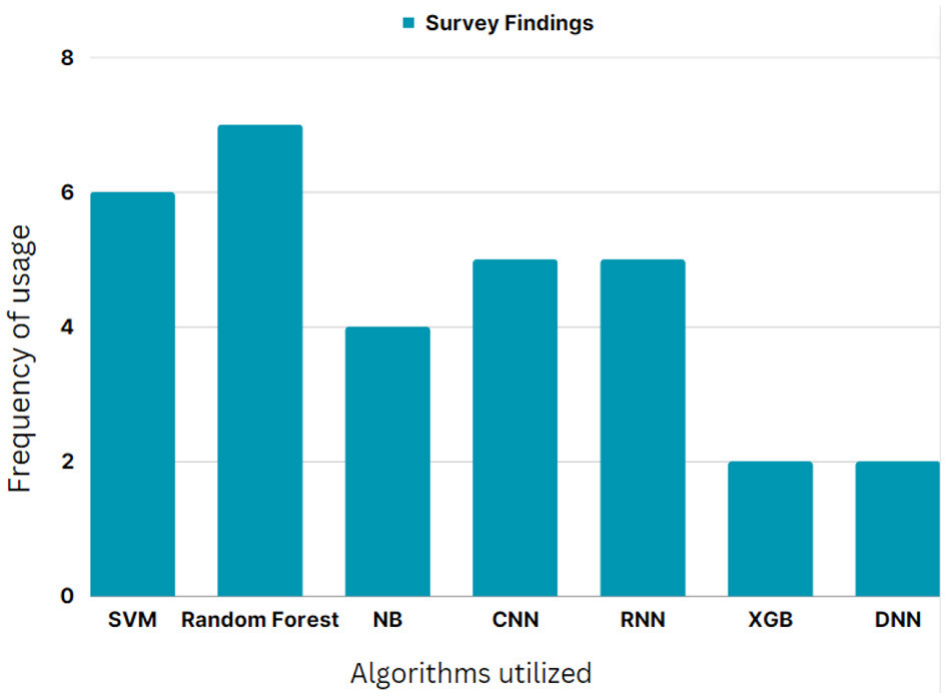
Most frequently used algorithms for text classification to detect cyberbullying.

The intelligent algorithms frequently presented by various authors in their prior works in detecting cyberbullying were consolidated and depicted in the form of a graph in Figure 1. From the graphical results, random forest is one of the majorly used ML approaches, while both CNN and RNN DL models made equal benchmarks in classification tasks.

## 3 Proposed framework

In this section, the datasets utilized for the study, the pre-processing steps involving NLP and vectorization techniques, feature engineering for balancing the datasets (Bahassine et al., 2020) and the overall workflow of the proposed methodology are explained in detail. Furthermore, the novel CNN-RF architecture proposed for text classification is proposed along with algorithmic implementation specifications. The comparative analysis of the implementation results is discussed in the next section.

### 3.1 Datasets

According to Chelmis and Zois (2021), Instagram is one of the most alarming social networking sites, accounting for approximately 25% of cyberbullying incidents worldwide. As a result, an effort was undertaken to create a sizable real-world Instagram dataset with approximately 4 million users and 10 million comments. The dataset was gathered by the authors via a snowballing technique. After being systematically categorized, this large volume of data was divided into subgroups to look for imbalanced elements. This led to the augmentation of the dataset subgroup with 10K comments that human experts carefully annotated. The Instagram 10K dataset obtained from Chelmis and Yao (2019) contains 40% of the obscene behavioral activity and 22.1% of the media sessions from the entire dataset. The dataset consists of 3 attributes, namely, *idx*, comment and label. The comment and label consist of textual data along with a predefined classification stating whether the comment can be termed bullying or not. The *idx* attribute specifies the post index on which all the mentioned comments are made. It shows that the dataset specifically deals with comments from 204 Instagram posts.

**Figure 2:**
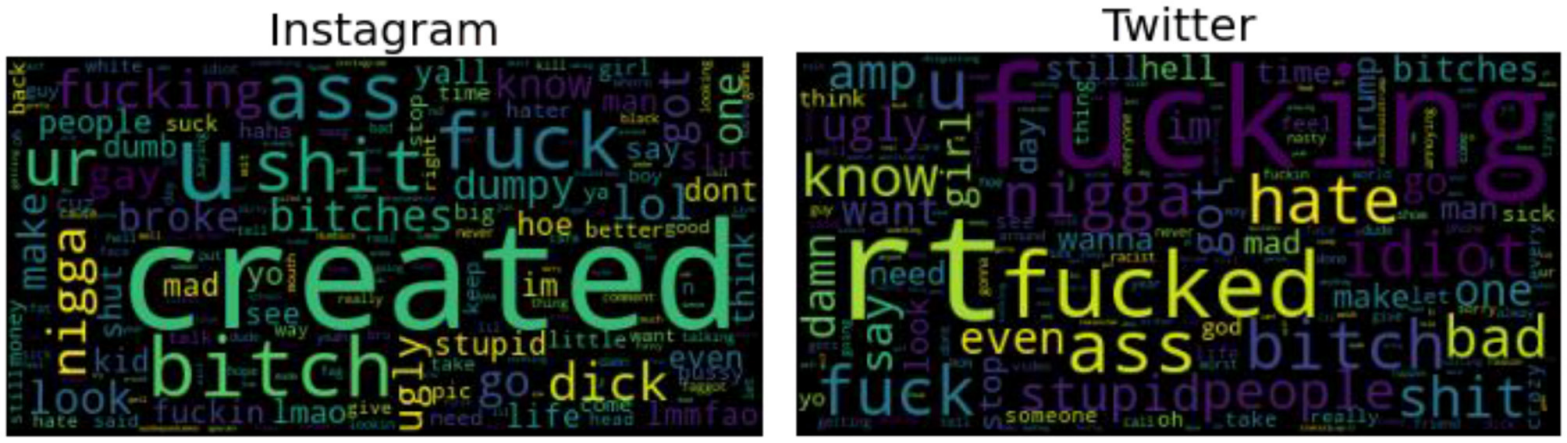
Word clouds of Instagram and Twitter datasets (bullying label).

The Twitter dataset used to test the proposed model is a massive dataset with 100K real-time tweets, in contrast to the small-scale Twitter datasets used by all previous academics for cyberbullying detection. Based on their 8 months of in-depth research, Founta et al. (2018) used incremental crowdsourcing methods to gather and annotate these data. The dataset consists of huge volumes of noise, making it difficult to deal with compared to the Instagram dataset. Given the poorly organized comments in this dataset, much pre-processing will be required before the dataset can be used to train any intelligent model. The word clouds of the Instagram and Twitter datasets after undergoing appropriate NLP pre-processing are shown in Figure 2.

### 3.2 Pre-processing

The datasets utilized for the purpose of this study were real-time and consist of massive amounts of noise inherent to the dynamic nature of various social media platforms. Before applying multivariable analysis techniques (Ochoa et al., 2011), a rigorous data preprocessing stage was implemented to ensure the quality and reliability of the dataset. This involved cleaning and filtering the social media content to remove noise, irrelevant information, and duplicate posts. Additionally, we conducted text normalization, stemming, and removal of stop words to standardize the textual data. The model inclusively addresses text-based classification for identifying cyberbullying episodes, and NLP is widely utilized to bolster the data pre-processing phase (Rosa et al., 2018). The datasets were initially converted into data frames, making it easier to perform modifications (Ahmed M. T. et al., 2021). The Instagram dataset, which consisted of an additional column depicting the post index, was modified to remove the “idx” column because it did not add any additional value to the classification task (Alam et al., 2021). The labels “yes” and “no” were converted into 1 and 0, respectively. The same replacement was done for the Twitter dataset as well. These modified datasets are then cleaned and pre-processed to convert the high-level human language into machine-understandable encodings (Dzisevic and Sesok, 2019). For data cleaning and feature extraction purposes, the datasets are passed on to six independent NLP functions. These include case conversion, removal of stop words, removal of special notations, removal of hypertext links and normalization of unnecessary spaces found in the textual content (Amali and Jayalal, 2020). The data obtained as the output from these steps contain less noise and are easier to handle for forming the encodings further (Ansary, 2020).

**Figure 3:**
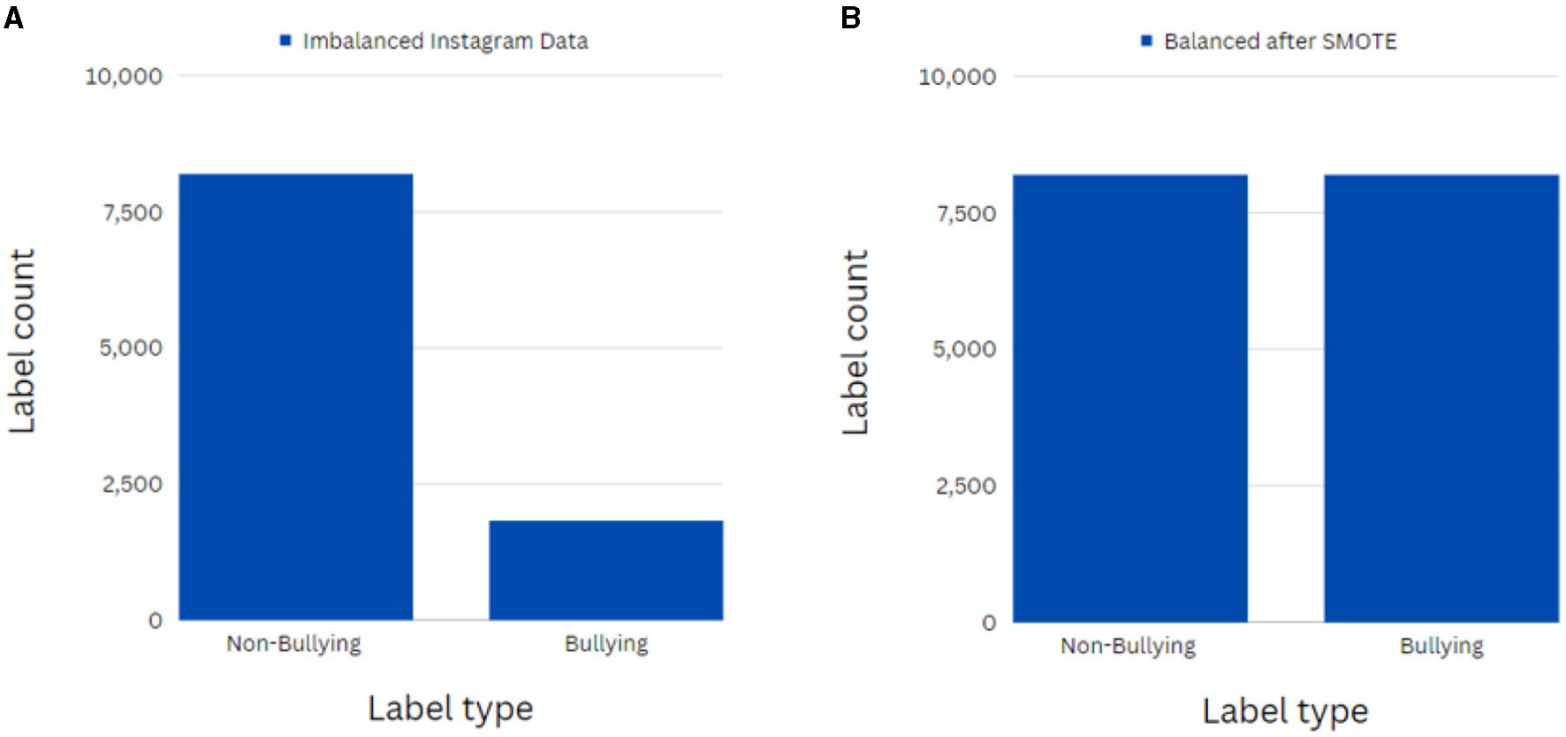
**(A)** Depiction of imbalanced class instances in the Instagram dataset. **(B)** Depiction of balanced class instances in the Instagram dataset after applying SMOTE.

Following this, a bar graph was used to understand the distribution of labels in the datasets. The Twitter dataset consisted of 53,852 false and 32,115 true class instances. Such major imbalance will critically affect the models' performances on the minority classes, thereby reducing the overall feasibility of these models (Gencoglu, 2021). Hence, this calls for an effective resampling technique to balance these datasets. The synthetic minority oversampling technique (SMOTE) oversampling algorithm was adopted to reduce the skew in the class distribution to 1:1 (Chawla et al., 2002). This algorithm synthesizes new cases in the minority class, it is the cyberbullying class instances by data augmentation and equalizes the number of instances in both classes. Figures 3A, B depict the working of this algorithm on the imbalanced Instagram dataset.

The steps discussed can be applied commonly to any form of tabular, textual data. The next step of pre-processing is based on the usage of encoding techniques for the conversion of datasets. These cleaned and balanced datasets must be converted into vectors and word encodings to apply the models. Since the output labels were already transformed into 0 s and 1 s during the prior cleaning procedures, the comments attributes should be transformed now. For traditional machine learning models, the TF-IDF vectorization statistical approach was adopted for comment transformations. In comment c, let x be the number of appearances of the word w. The term (TF) is calculated as shown in Equation 1, wherein the summation term in the denominator specifies the occurrence of the given term over all the comments in the dataset.


(1) $$\begin{array}{l} TF=\;x\;/\;\sum \;xi \end{array}$$

Then, inverse document frequency (IDF) is calculated by dividing the total number of comments by the number of comments consisting of the term w. The log of this value will provide the IDF, which is mentioned in Equation 2. Multiplying the TF and IDF terms obtained from these equations will give the TF-IDF value of word w, as shown in Equation 3.


(2) $$\begin{array}{l} IDF=log\left(\frac{C}{\left[Ci:w\in C\right]}\right) \end{array}$$

(3) $$\begin{array}{l} T\;F-IDF=\left(TF\;*\;IDF\right) \end{array}$$

For Neural Network models, GloVe (Global Vectors) has been used to perform these word transformations (Ziems et al., 2020). It is an established unsupervised learning approach for creating word embeddings, which are vector representations of words in a high-dimensional space and uses word co-occurrence data from a corpus to learn about the semantic and syntactic links between words (Wang et al., 2021). This method measures the frequency of word pairings that appear within a given frame of text by creating a co-occurrence matrix. Then, using matrix factorization techniques, a collection of word embeddings is learned from the elements in this matrix. The resulting vectors express each word as a high-dimensional vector, with each dimension's values denoting the degree to which the word and a given context are related to one another. For instance, words such as “Man” and “Father” display high cosine similarity. Let v_i_ and v_j_ be the vectors of the words w_i_ and w_j_, respectively. The correlation between these words along with their probe words is calculated as shown in Equation 4, where v_k_ stands for the probe words and P_ik/jk_ depicts the probability of co-occurrence of these words.


(4) $$\begin{array}{l} F\left(v_{i},v_{j},v_{k}\right)=P_{ik}/P_{jk} \end{array}$$

**Figure 4:**
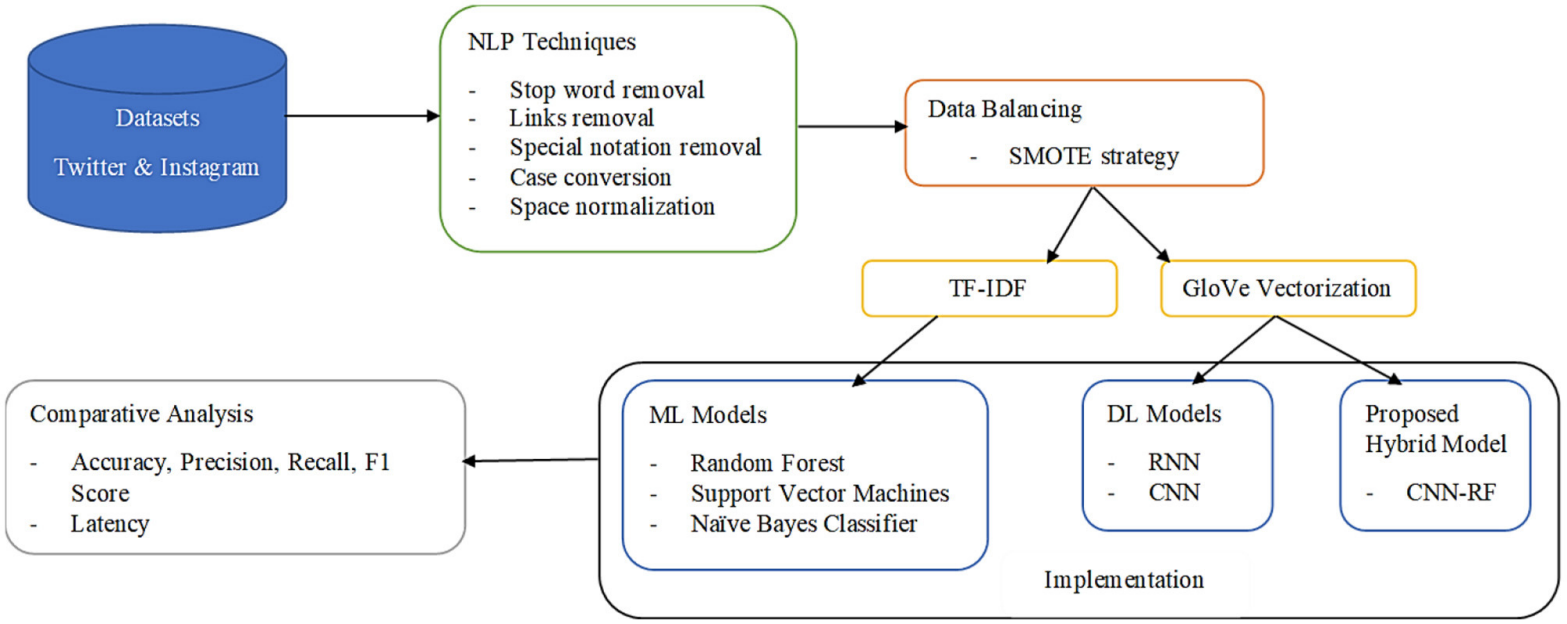
Methodology of the proposed framework.

The transformed textual data are split into training and testing in a 70:30 ratio by preserving the class distribution of the balanced original dataset (Akhter et al., 2023). These were then applied to different preexisting intelligent algorithms along with the novel CNN-RF model proposed in this study. The workflow of the proposed framework is displayed in Figure 4. The performance of each of these algorithms on the selected data is elucidated in the next section.

### 3.3 Proposed model

The proposed model is aimed at providing faster execution with accurate results across different social media platforms. Moreover, deep learning algorithms work quite well on massive datasets but suffer from the problem of overfitting when less data are available (Al-Garadi et al., 2019). The proposed CNN-RF model works to solve this issue by combining the goodness of both ML and DL concepts. The initial model analysis with traditional machine learning models such as RF, SVM, and naïve Bayes suggested in prior studies has been applied independently to both datasets. The higher accuracy obtained by the RF model, irrespective of the difference in the sizes of these datasets, acted as a strong motivation to adopt this model further. Among the DL models, CNN models also showed a similar pattern of consistency across both datasets. To capture the nuanced features indicative of cyberbullying, a comprehensive set of features was extracted from the preprocessed data. This included linguistic features such as sentiment polarity, frequency of offensive words, and syntactic features like sentence length and grammatical structure. Social network features, such as user interactions and network centrality, were also incorporated to capture the contextual dynamics of online communication.

CNNs are specialized neural networks that are used for image recognition and processing (Lu et al., 2020). CNN applies a collection of convolutional layers to the source image, applying filters to the image to extract features such as borders, texturing, and forms. Each convolutional layer's output is then processed through a non-linear activation function, such as Rectified Linear Unit (ReLU) (Equation 5), to provide the network with non-linearity and help it learn more intricate patterns (Umer et al., 2023). However, CNNs can perform equally well on text classification. Text-based CNNs work on word embeddings in the form of matrices instead of conventional image pixels. Hence, these operate with one -dimensional convolution layers (Ahmed M. F. et al., 2021).


(5) $$\begin{array}{l} F\left(n\right)\;=\;max\left(0,n\right) \end{array}$$

The input word embeddings are in the form of a matrix, as mentioned above. This can be represented as Equation 6 with I being the input, n being the number of words in the input comment, d being the dimension of the matrix and R∧ notation denoting that the matrices are real-valued.


(6) $$\begin{array}{l} I=R^{\wedge }\;\left(n\;*\;D\right) \end{array}$$

Next, a predetermined number of convolutional filters are applied to the input. For the purposes of this study, this parameter is set to an ideal value of 32 1D filters. A feature map of the selected filters is generated as a result. If l is the length of the filters used, the output may be represented as Equation 7. This output layer's rows each correspond to a word or local pattern that the filter extracts from the input text. This operation by the convolutional model can be shown as Equation 8, where b ∈ R∧(F) is the bias, W ∈ R∧(l^*^D) is the weight, F is the number of filters and r stands for the ReLU function.


(7) $$\begin{array}{l} O\;\in \;R^{\wedge }\left(n-l+1\;*\;F\;\right) \end{array}$$

(8) $$\begin{array}{l} O\text{\_}i\;=\;f\;\left(W\cdot \;I\left[x:x+l-1\right]\;+\;b\right) \end{array}$$

The resulting output feature maps are then reduced (O_r_) in size using a small window that runs over a few rows at a time to perform the max-pooling operation. The reduced output map can be depicted as Equation 9, where s stands for the size of the pooling window. The overall mathematical operation can be formed as Equation 10.


(9) $$\begin{array}{l} O_{r}\;\in \;R^{\wedge }\left(\left(n-l+1/s\right)\;*\;K\right) \end{array}$$

(10) $$\begin{array}{l} O_{i,j}=\;max\left(O\left[is:\left(i+1\right)s,\;j\right]\right) \end{array}$$

Finally, to finish the classification process, the required number of fully connected layers may be applied to the pooling layer's output. An output probability distribution across the potential output classes results from the last fully linked layer.

**Figure 5:**
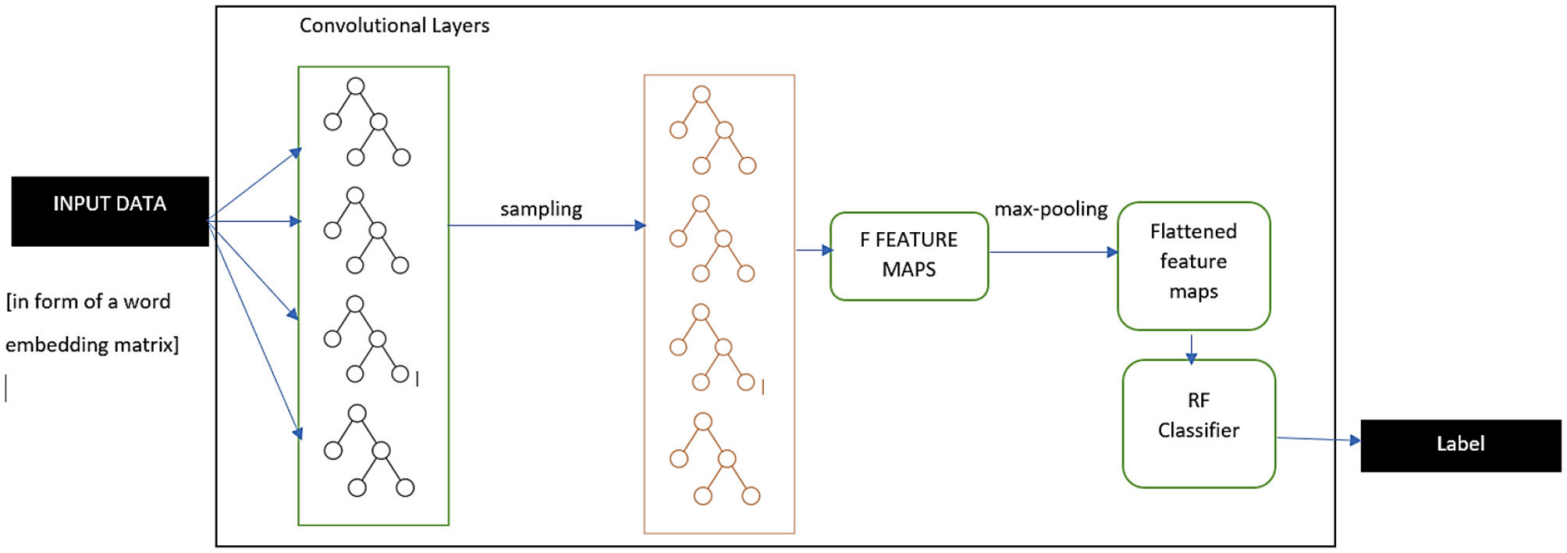
CNN-RF model working architecture.

The above discussed model marks the overall working principle of individual CNN algorithms for performing text classification. For the proposed hybrid CNN-RF model, slight changes were made to the final steps where the model exquisitely deals with fully connected layers. The overall working type of the proposed algorithm is displayed in Figure 5. After reduction of the output feature map using the pooling functionality, the obtained reduced outcome is flattened further into a one-dimensional vector. This is passed on to the traditional RF model, which performs reliable and robust classification tasks with the help of multiple decision trees. The details of this classifier are explained in Section 4. Equation 11 specifies the final step in the CNN-RF model taking Y as the class label that is being predicted and RF as the classification model.


(11) $$\begin{array}{l} Y\;=\;RF\left(O_{r}\right) \end{array}$$

Using stochastic gradient descent and backpropagation, the parameters of the CNN-RF model including the filter weights, bias terms, and fully connected layer weights are trained to reduce the discrepancy between the model's expected and actual output.

The algorithm for this novel textual CNN-RF model has been mentioned as Algorithm 1. This model is implemented on the selected real-time Instagram and Twitter datasets, and its performance is evaluated against five independent and top-tier text classification approaches utilized among ML and DL technologies. The results of this model are studied in detail in the next section, and its future prospects are proposed to pave the way for socially conscious objectives.

**Algorithm 1:**
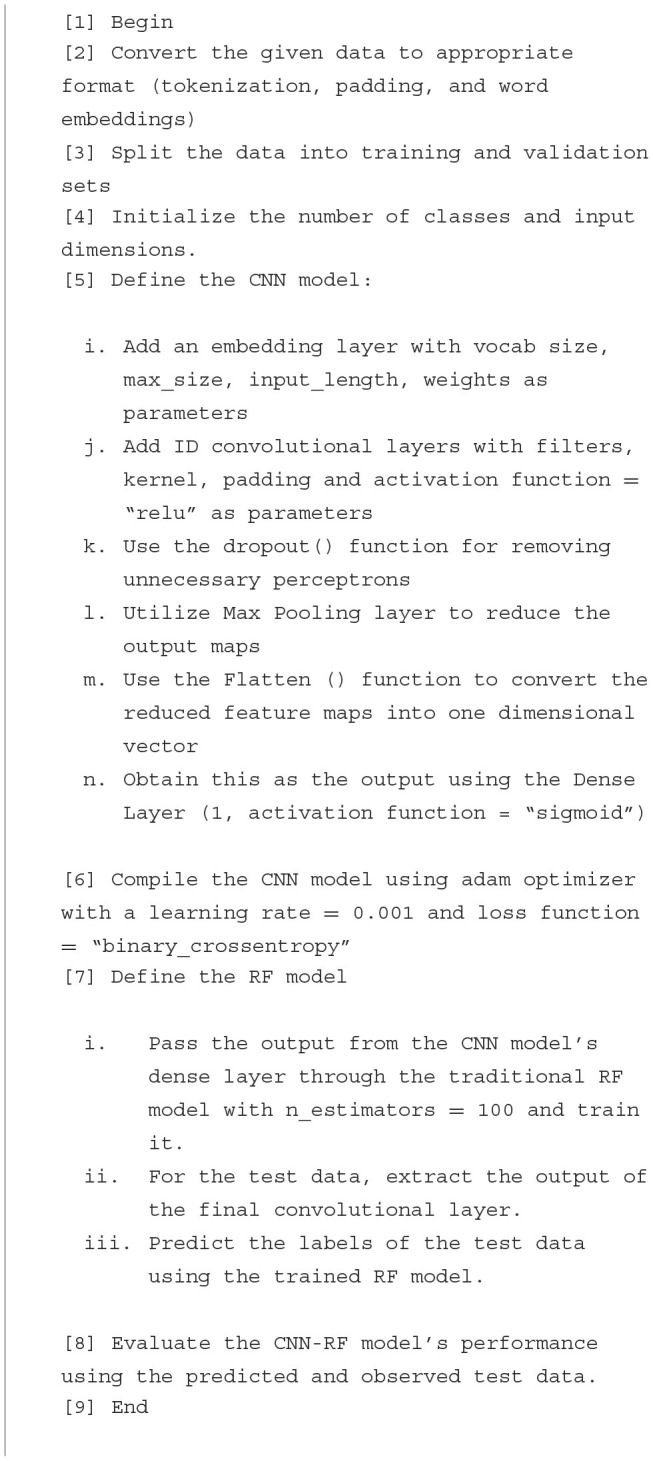
CNN-RF for text classification.

## 4 Results and discussion

This section deals with the comparative analysis of the results obtained from the implementation of various algorithms on selected datasets. The primary parameters utilized for carrying out this analysis are accuracy, recall, precision and F1-score (Zulqarnain et al., 2020; Gupta et al., 2021). Along with these, an additional factor of decrease in execution latency has been demonstrated while using the novel CNN-RF model for text classification.

The evaluation metrics used for analyzing the performance of the models depend upon four primary values namely,

- True Positives (TP): The measure of instances that the classifier properly classifies as positive and that are genuinely positive.
- True Negatives (TN): The number of cases that the classifier properly classifies as negative and are truly negative.
- False Positives (FP): When a classifier incorrectly classifies an instance as being in the positive class when it is actually in the negative class.
- False Negatives (FN): When the classifier classifies an instance as positive even if it belongs to the negative class.

In binary classification, true positives and true negatives are crucial performance indicators because they show how well the classifier accurately identifies instances that belong to the positive and negative classes, respectively. They are also used to determine additional performance metrics, including the F1 score, recall, and precision. Their respective derivations from these four mentioned values are shown in Equations 12–15.


(12) $$\begin{array}{l} Accuracy=\frac{TP+TN}{TP+TN+FP+\;FN} \end{array}$$

(13) $$\begin{array}{l} Precision=\frac{TP}{TP+FP} \end{array}$$

(14) $$\begin{array}{l} Recall=\frac{TP}{TP+FN} \end{array}$$

(15) $$\begin{array}{l} F-score=\frac{2\;*\;precision\;*\;recall}{precision+recall} \end{array}$$

The accuracy of a classifier is measured by how well it predicts the proper class for each instance in the dataset. The number of accurate predictions divided by the total number of predictions is used to compute it. Precision is the percentage of positive cases that were accurately detected among all instances that the classifier expected to be positive. It is measured as the proportion of actual positive results to all cases that were projected to be positive. The F1 score, which is a harmonic mean of precision and recall, is an effective way to balance the trade-off between them. The weighted average of precision and recall is used to determine the F1 score. Higher values of the classifier's accuracy, precision, recall, and F1 score generally imply greater performance, whereas lower values often suggest poorer performance.

The balanced and transformed datasets after undergoing the train-test split are applied to ML approaches. These approaches were chosen and finalized based on prior research studies suggesting the superior performance of these models in terms of binary text classification tasks. The model generates a substantial number of decision trees by optimizing the information gained at each node and by dividing the data depending on given attributes. In the final prediction stage, the result is calculated by averaging the results from all the trees in the forest (Kim et al., 2021). The ensemble computation of the RF model is specified in Equation 16. The RF approach provides several benefits, including managing missing values and noisy data, avoiding overfitting, and increasing the generalization performance of the model.


(16) $$\begin{array}{l} F\left(x\right)=\;\overset{N}{\underset{1}{\sum }}\frac{1}{N}\;F_{n}\left(x\right) \end{array}$$

Next, the naïve Bayes model was implemented. This is a probabilistic supervised learning approach that works with a likelihood function that illustrates the probability of witnessing a specific value of a feature. It assumes that, given the class, the features are conditionally independent of one another (Muneer and Fati, 2020). For balanced classes, the prior probability is assumed to be equal. The Bernoulli distribution is employed for binary classification in this study, as indicated in Equation 17, where μ_ic_ stands for the likelihood that feature *i* occurs in class *c* . This model is computationally effective since it can be trained quickly on huge datasets, but its conditional independence presumption can sometimes be unduly simple, resulting in inferior performance when the characteristics are significantly coupled.


(17) $$\begin{array}{l} P\left(u|v=c,\;\theta \right)=\;\overset{N}{\underset{i=1}{\prod }}Bern\left(u_{i}|\mu_{ic}\right) \end{array}$$

Finally, the SVM classifier is implemented. Its mathematical theory entails finding the best hyperplane that maximizes the margin between the various classes of data points, applying an optimization algorithm to solve a constrained optimization problem, and when necessary, transforming the data into a higher-dimensional space using a kernel function (Unni et al., 2021; Sainju et al., 2022). Kernel function selection is crucial since it affects how the decision border is shaped. The most popular kernel function utilized for dynamic classification purposes is the radial basis function (RBF), which is depicted in Equation 18, wherein u and v are data points in the input space whose Euclidean distance is calculated and multiplied by ɤ, the shape controlling parameter.


(18) $$\begin{array}{l} F\left(u,v\right)=\exp\left(-\gamma {\left||u-v|\right|}^{2}\right) \end{array}$$

Among these ML models, the RF model performed the best, giving the highest accuracy for both datasets. Furthermore, the two most utilized DL models are implemented independently on both datasets, and their evaluation metrics are obtained. The fundamental working principles of the CNN model have already been discussed in Section 3. The RNN model, on the other hand, is a form of neural network created to handle sequential data and depends on both the current input and the prior inputs and outputs.

The LSTM employs a memory cell with long-term information storage capacity and three gates (input, forget, and output) that control the flow of data into and out of the cell. Backpropagation through time (BPTT), a backpropagation algorithm variation used to update the weights and biases of the network, is used to train RNNs (Du et al., 2021). Because of the backpropagation of the gradient at each time step over the whole network, training is computationally costly and vulnerable to the vanishing gradient problem. At time *T*, in network *N*, let the input be An. The previous time is T-1, for which the cell state is *C*(*t*−1) and the hidden state is *H*(*t*−1). Using these forget gates computes the amount of information from previous cells that should be retained within using Equation 19. The input gate calculates the amount of new information to be introduced into the cell state using Equation 20. The given new information to be added is depicted by the candidate cell state using Equation 21, and the current cell state gives the combination of the candidate state and previous states using Equation 22.


(19) $$\begin{array}{l} FG=sigmoid\;\left(W_{f\;}*\left[H\left(t-1\right),\;A_{n}\right]+\;b_{f}\right) \end{array}$$

(20) $$\begin{array}{l} FG=sigmoid\;\left(W_{i\;}*\left[H\left(t-1\right),\;A_{n}\right]+\;b_{i}\right) \end{array}$$

(21) $$\begin{array}{l} Can\text{\_}C\;=tanh\;\left(W_{c\;}*\left[H\left(t-1\right),\;A_{n}\right]+\;b_{c}\right) \end{array}$$

(22) $$\begin{array}{l} CC\;=FG*C\left(t-1\right)+IG\;*\;Can\text{\_}C \end{array}$$

The output gate is the amount of the current state to be given to the hidden state as output based on the given input and previous states. This is computed as Equation 23. Finally, the current hidden state is obtained from the refinement of the current cell state through the output gate, as shown in Equation 24. The [*H*(*t*−1), *An*] represents the combination of the current input and prior hidden state. *Wf, Wi, Wc, Wo* and *bf, bi, bc, bo* represent the weights and bias vectors, respectively.


(23) $$\begin{array}{lll} OG & = & sigmoid\;\left(W_{o\;}\;*\;\left[H\left(t-1\right),\;A_{n}\right]+\;b_{o}\right) \end{array}$$

(24) $$\begin{array}{lll} HG\; & = & OG\;*\;tanh\;\left(CC\right) \end{array}$$

Of the two DL models, the CNN model provided better precision and accuracy scores than the RNN model. The CNN's accuracy was almost 5 points above the RNN accuracy in both cases. This further gave a strong reason for considering CNN as the base model to be combined with the RF model from traditional ML approaches. Thus, the novel CNN-RF algorithm for text- classification was adopted and implemented on the chosen datasets.

The evaluation metrics of each of the implemented models are specified in Tables 1, 2 for the Instagram and Twitter datasets, respectively. It can be clearly noticed that the RF model outperforms the SVM and naïve Bayes models among traditional ML approaches with overall accuracies of 0.83 and 0.94, respectively, compared to 0.80 and 0.92 accuracy scores obtained by SVM, which performed the second best. The RNN model obtained accuracies of approximately 0.85 and 0.89, while the CNN model gave higher accuracies of approximately 0.89 and 0.93 for the Instagram and Twitter datasets, respectively. However, the proposed CNN-RF model outperforms all the independent techniques by acquiring an accuracy score of 0.96 and a precision of approximately 0.98 for Instagram data and an accuracy score of 0.98 and a precision of approximately 0.99 for Twitter data.

**Table 1:** Performance parameters of the suggested models applied over the Instagram dataset.

**Model**	**Accuracy**	**Precision**	**Recall**	**F1-Score**
RF	0.83	0.87	0.74	0.80
SVM	0.80	0.83	0.76	0.79
Naïve bayes	0.78	0.79	0.76	0.77
RNN	0.8462	0.89	0.8544	0.8695
CNN	0.8921	0.9122	0.872	0.8916
RF-CNN	0.9586	0.9881	0.9267	0.9564

**Table 2:** Performance parameters of the suggested models applied over the Twitter dataset.

**Model**	**Accuracy**	**Precision**	**Recall**	**F1-Score**
RF	0.94	0.93	0.95	0.94
SVM	0.92	0.88	0.97	0.92
Naïve bayes	0.89	0.88	0.90	0.89
RNN	0.8923	0.9232	0.85	0.8850
CNN	0.9344	0.9510	0.9021	0.9259
RF-CNN	0.9841	0.9971	0.9419	0.9687

Table 1 displays a comparative assessment between two distinct models, specifically Word2Vec and FastText, utilizing three different classifiers: Random Forest (RF), Support Vector Machine (SVM), and Naïve Bayes. The metrics employed for performance evaluation encompass Accuracy, Precision, Recall, and F1-Score. For the Word2Vec model, the achieved accuracy is 71.05% with RF, 69.72% with SVM, and 58% with Naïve Bayes. The corresponding precision values are 0.75, 0.69, and 0.56, while recall values stand at 0.71, 0.69, and 0.58. F1-Scores for Word2Vec with RF, SVM, and Naïve Bayes are 0.67, 0.69, and 0.48 respectively.

In contrast, the FastText model demonstrates an accuracy of 79.05% with RF, 82% with SVM, and 55.69% with Naïve Bayes. Precision values for FastText are 0.8, 0.81, and 0.57, and recall values are 0.78, 0.81, and 0.56. F1-Scores for FastText with RF, SVM, and Naïve Bayes are 0.79, 0.80, and 0.55 respectively. The data implies that, overall, the FastText model surpasses the Word2Vec model in terms of accuracy, precision, recall, and F1-score when applied to RF and SVM classifiers. However, both models exhibit comparatively lower performance when employed with the Naïve Bayes classifier (Tables 3, 4).

**Table 3:** Comparative performance analysis of Word2Vec and FastText models.

**Model**		**RF**	**SVM**	**Naïve bayes**
Word2Vec	Accuracy	71.05%	69.72%	58%
	Precision	0.75	0.69	0.56
	Recall	0.71	0.69	0.58
	F1-Score	0.67	0.69	0.48
FastText	Accuracy	79.05%	82%	55.69%
	Precision	0.8	0.81	0.57
	Recall	0.78	0.81	0.56
	F1-Score	0.79	0.8	0.55

**Table 4:** Execution times of suggested models for both datasets.

**Models**	**Execution time - Instagram (in seconds)**	**Execution time- Twitter (in seconds)**
RF	55.6	56.2
SVM	45.8	62.1
NB	34.1	59.33
RNN	98.3	127.2
CNN	33.2	55.1
RF-CNN	29.8	51.75

Furthermore, the execution latencies of each of these algorithms were considered to facilitate faster aid to prevent cyberbullying incidents and serve bullied victims immediately. The RNN model took comparatively more time to process the data, while the CNN worked relatively faster with an optimal learning rate. ML models are executed quite quickly on these massive datasets. The overall concatenation of the CNN and RF models decreased the model training time further by almost 3.4 seconds compared to the fastest models in the case of both datasets. This further proves the efficiency and reliability of this model for application in real-time detection scenarios.

Combining the findings, all the standard indicators show good performance. While the RF and CNN models separately withered to 83% and 89%, respectively, in the case of the Instagram dataset, the accuracy is above 95% for the combined deep learning CNN-RF technique. A similar pattern is observed with the Twitter dataset as well, although the accuracy gap between the CNN-RF model and separate RF, CNN models is noticeably less. This suggested that, in contrast to smaller datasets such as the Instagram dataset, classical machine learning algorithms performed much better on big datasets. In smaller datasets, neural network models fared better than ML techniques (Sainju et al., 2022). Nonetheless, compared to RNN or other conventional ML models, the CNN model achieved higher accuracy in both cases. The CNN network's parametric values, including neuron count, dense layer scale, and dropout rates, led to excellent results in the experiments. As a result, the same basic parametric choices were also applied to the suggested CNN-RF model, resulting in an approach that is more effective and quicker to handle datasets of any size.

## 5 Conclusion and future work

Cyberbullying is a severe issue that can manifest itself in a variety of ways, including posting hurtful remarks or pictures, threatening messages, disseminating false information, or adopting someone's identity online. It affects many individuals, especially children and teens who use social media or participate in online forums, and it has major psychological and emotional repercussions for its victims, including depression, low self-esteem, suicidal thoughts, and distrustful attitudes (Ansary, 2020). This study aims to address this issue by examining and demonstrating the best algorithms for real-time social data. An extensive survey was carried out to identify the finest approaches accessible under classical ML and DL algorithms for text-based cyberbullying. Additionally, a cutting-edge hybrid CNN-RF method was created to combine the benefits of strong, independent ML and DL models. This ensured that the model was impartial to the dataset sizes and consistently produced the best results. The purpose of adopting two real-world datasets from two separate platforms with varying size scales was to demonstrate the compatibility of the novel algorithm for textual classification across different platforms.

All general metrics showed that the CNN-RF model performed the best, producing the added benefit of a faster execution time, making it a trustworthy source to be used in real-time social media applications' dynamic data settings for quicker bullying incident identification. The CNN-RF model used in this work uses text-based classification to identify online bullying. Given that CNN-RF models are notoriously used for picture prediction tasks by subject-matter experts, the same hybrid approach may be readily expanded to image- or video-based material in the future (Ge et al., 2021; Kumari and Singh, 2021). Moreover, these detection models may be integrated to create a complete multimodal system (Qiu et al., 2022) that can quickly and effectively identify bullying episodes that take place online in any format. These models can be connected to social media software to aid with improved defense mechanisms, which will help lessen the harm caused by these online bullying incidents.

## Data availability statement

The datasets presented in this study can be found in online repositories. The names of the repository/repositories and accession number(s) can be found below: https://github.com/t-davidson/hate-speech-and-offensive-language.

## Author contributions

DB: Conceptualization, Investigation, Writing—review & editing. TN: Conceptualization, Data curation, Methodology, Writing—original draft. SS: Conceptualization, Formal analysis, Investigation, Supervision, Validation, Writing—review & editing. MP: Formal analysis, Validation, Writing—review & editing. ES: Conceptualization, Validation, Visualization, Writing—review & editing. NG: Data curation, Visualization, Writing—review & editing. LU: Validation, Visualization, Writing—review & editing.

## References

[B1] Ahmed M. F. Mahmud Z. Biash Z. T. Ryen A. A. N. Hossain A. Ashraf F. B. . (2021). Cyberbullying detection using deep neural network from social media comments in bangla language. arXiv preprint arXiv:2106.04506.

[B2] Ahmed M. T. Rahman M. Nur S. Islam A. Das D. (2021). “Deployment of machine learning and deep learning algorithms in detecting cyberbullying in bangla and romanized bangla text: a comparative study,” in 2021 International Conference on Advances in Electrical, Computing, Communication and Sustainable Technologies (ICAECT) (IEEE), 1–10. 10.1109/ICAECT49130.2021.9392608

[B3] Akhter A. Acharjee U. K. Talukder M. A. Islam M. M. Uddin M. A. (2023). A robust hybrid machine learning model for Bengali cyber bullying detection in social media. Nat. Lang. Proc. J. 4:100027. 10.1016/j.nlp.2023.100027

[B4] Alam K. S. Bhowmik S. Prosun P. R. K. (2021). “Cyberbullying detection: an ensemble based machine learning approach,” in 2021 Third International Conference on Intelligent Communication Technologies and Virtual Mobile Networks (ICICV) (IEEE), 710–715. 10.1109/ICICV50876.2021.9388499

[B5] Al-Garadi M. A. Hussain M. R. Khan N. Murtaza G. Nweke H. F. Ali I. . (2019). Predicting cyberbullying on social media in the big data era using machine learning algorithms: review of literature and open challenges. IEEE Access 7, 70701–70718. 10.1109/ACCESS.2019.2918354

[B6] Amali H. I. Jayalal S. (2020). “Classification of cyberbullying Sinhala language comments on social media,” in 2020 Moratuwa Engineering Research Conference (MERCon) (IEEE), 266–271. 10.1109/MERCon50084.2020.9185209

[B7] Ansary N. S. (2020). Cyberbullying: Concepts, theories, and correlates informing evidence-based best practices for prevention. Aggress. Violent Behav. 50:101343. 10.1016/j.avb.2019.101343

[B8] Bahassine S. Madani A. Al-Sarem M. Kissi M. (2020). Feature selection using an improved Chi-square for Arabic text classification. J. King Saud Univ. - Comput. Inf. Sci. 32, 225–231. 10.1016/j.jksuci.2018.05.010

[B9] Banerjee V. Telavane J. Gaikwad P. Vartak P. (2019). “Detection of cyberbullying using deep neural network,” in 2019 5th International Conference on Advanced Computing and Communication Systems (ICACCS) (Coimbatore, India: IEEE), 604–607. 10.1109/ICACCS.2019.8728378

[B10] Chawla N. V. Bowyer K. W. Hall L. O. Kegelmeyer W. P. (2002). SMOTE: synthetic minority over sampling technique. J. Artif. Intell. Res. 16, 321–357. 10.1613/jair.953

[B11] Chelmis C. Yao M. (2019). “Minority report: cyberbullying prediction on Instagram,” in Proceedings of the 10th ACM Conference on Web Science (Boston Massachusetts USA: ACM), 37–45. 10.1145/3292522.3326024

[B12] Chelmis C. Zois D. S. (2021). Dynamic, incremental, and continuous detection of cyberbullying in online social media. ACM Trans. Web. 15, 1–33. 10.1145/3448014

[B13] Chia Z. L. Ptaszynski M. Masui F. Leliwa G. Wroczynski M. (2021). Machine Learning and feature engineering-based study into sarcasm and irony classification with application to cyberbullying detection. Inf. Process. Manag. 58:102600. 10.1016/j.ipm.2021.102600

[B14] Du J. Vong C. M. Chen C. L. P. (2021). Novel efficient RNN and LSTM-like architectures: recurrent and gated broad learning systems and their applications for text classification. IEEE Trans. Cybern. 51, 1586–1597. 10.1109/TCYB.2020.2969705 32086231

[B15] Dzisevic R. Sesok D. (2019). “Text classification using different feature extraction approaches,” in 2019 Open Conference of Electrical, Electronic and Information Sciences (eStream) (Vilnius, Lithuania: IEEE), 1–4. 10.1109/eStream.2019.8732167

[B16] Founta A. Djouvas C. Chatzakou D. Leontiadis I. Blackburn J. Stringhini G. . (2018). “Large scale crowdsourcing and characterization of twitter abusive behavior,” in Proceedings of the International AAAI Conference on Web and Social Media. 10.1609/icwsm.v12i1.14991

[B17] Ge S. Cheng L. Liu H. (2021). “Improving cyberbullying detection with user interaction,” in Proceedings of the Web Conference 2021 (Ljubljana Slovenia: ACM), 496–506. 10.1145/3442381.3449828

[B18] Gencoglu O. (2021). Cyberbullying detection with fairness constraints. IEEE Internet Comput. 25, 20–29. 10.1109/MIC.2020.3032461

[B19] Gummadavelly H. Goud K. P. Reddy S. K. K. Ramchander N. S. (2021). Cyber bullying detection using machine learning. Int. J. Emerg. Technol. Innov. Res. 8, b535–b540.

[B20] Gupta D. Khanna A. Kansal V. Fortino G. Hassanien A. E. (2021). “Proceedings of Second Doctoral Symposium on Computational Intelligence: DoSCI,” in Advances in Intelligent Systems and Computing (Singapore: Springer Singapore), 1374. 10.1007/978-981-16-3346-1

[B21] Hosseinmardi H. Mattson S. A. Ibn Rafiq R. Han R. Lv Q. Mishra S. . (2015). Detection of cyberbullying incidents on the Instagram social network. arXiv 2015.arXiv preprint arXiv:1503.03909.

[B22] Islam M. M. Uddin M. A. Islam L. Akter A. Sharmin S. Acharjee U. K. . (2020). “Cyberbullying detection on social networks using machine learning approaches,” in 2020 IEEE Asia-Pacific Conference on Computer Science and Data Engineering (CSDE) (Gold Coast, Australia: IEEE), 1–6. 10.1109/CSDE50874.2020.9411601

[B23] Kastrati Z. Imran A. S. Kurti A. (2019). Integrating word embeddings and document topics with deep learning in a video classification framework. Patt. Recogn. Lett. 128, 85–92. 10.1016/j.patrec.2019.08.019

[B24] Keni A. Kini M. (2020). “Cyber-bullying detection using machine learning algorithms,” in Computer Science, Psychology.

[B25] Kim S. Razi A. Stringhini G. Wisniewski P. J. De Choudhury M. (2021). A human-centered systematic literature review of cyberbullying detection algorithms. Proc. ACM Hum. Comput. Interact. 5, 1–34. 10.1145/347606636644216

[B26] Kumar A. Sachdeva N. (2022). A Bi-GRU with attention and CapsNet hybrid model for cyberbullying detection on social media. World Wide Web. 25, 1537–1550. 10.1007/s11280-021-00920-4

[B27] Kumar R. (2020). Detection of cyberbullying using machine learning. Int. J. Res. Appl. Sci. Eng. Technol. 8, 1231–1240. 10.22214/ijraset.2020.30403

[B28] Kumari K. Singh J. P. (2021). Identification of cyberbullying on multi-modal social media posts using genetic algorithm. Trans. Emerg. Telecommun. Technol. 32:e3907. 10.1002/ett.3907

[B29] López-Vizcaíno M. F. Nóvoa F. J. Carneiro V. Cacheda F. (2021). Early detection of cyberbullying on social media networks. Future Gener. Comput. Syst. 118, 219–229. 10.1016/j.future.2021.01.006

[B30] Lu N. Wu G. Zhang Z. Zheng Y. Ren Y. Choo K. K. R. . (2020). Cyberbullying detection in social media text based on character-level convolutional neural network with shortcuts. Concurr. Comput. Pract. Exp. 32:e5627. 10.1002/cpe.5627

[B31] Muneer A. Fati S. M. (2020). A comparative analysis of machine learning techniques for cyberbullying detection on Twitter. Fut. Internet 12:187. 10.3390/fi12110187

[B32] Murshed B. A. H. Abawajy J. Mallappa S. Saif M. A. N. Al-Ariki H. D. E. (2022). DEA-RNN: A hybrid deep learning approach for cyberbullying detection in Twitter social media platform. IEEE Access 10, 25857–25871. 10.1109/ACCESS.2022.3153675

[B33] Nirmal N. Sable P. Patil P. Kuchiwale S. (2021). Automated detection of cyberbullying using machine learning. Int. Res. J. Eng. Technol. 7, 2054–2061.

[B34] Ochoa A. Ponce J. Jaramillo R. Ornelas F. Hernandez A. Azpeitia D. . (2011). “Analysis of Cyber-bullying in a virtual social networking,” in 2011 11th International Conference on Hybrid Intelligent Systems (HIS) (IEEE), 229–234. 10.1109/HIS.2011.6122110

[B35] Pradhan A. Yatam V. M. Bera P. (2020). “Self-attention for cyberbullying detection,” in 2020 International Conference on Cyber Situational Awareness, Data Analytics and Assessment (CyberSA) (Dublin, Ireland: IEEE), 1–6. 10.1109/CyberSA49311.2020.9139711

[B36] Qiu J. Moh M. Moh T.-. S. (2022). “Multi-modal detection of cyberbullying on Twitter,” in Proceedings of the ACM Southeast Conference (ACM), 9–16. 10.1145/3476883.3520222

[B37] Raj C. Agarwal A. Bharathy G. Narayan B. Prasad M. (2021). Cyberbullying detection: hybrid models based on machine learning and natural language processing techniques. Electronics 10:2810. 10.3390/electronics10222810

[B38] Raj M. Singh S. Solanki K. Selvanambi R. (2022). An application to detect cyberbullying using machine learning and deep learning techniques. SN Comput. Sci. 3:401. 10.1007/s42979-022-01308-5 35911437 PMC9321314

[B39] Rosa H. Matos D. Ribeiro R. Coheur L. Carvalho J. P. (2018). “A ‘Deeper' look at detecting cyberbullying in social networks,” in 2018 International Joint Conference on Neural Networks (IJCNN) (Rio de Janeiro: IEEE), 1–8. 10.1109/IJCNN.2018.8489211

[B40] Sainju K. D. Young L. Kuffour A. Mishra N. (2022). A machine learning and qualitative examination of cyberbullying disclosures on Twitter. J. Soc. Media Soc. 11, 209–235.

[B41] Samghabadi N. S. (2020). Automatic detection of nastiness and early signs of cyberbullying incidents on social media. (Doctoral dissertation, University of Houston).

[B42] Umer M. Imtiaz Z. Ahmad M. Nappi M. Medaglia C. Choi G. S. . (2023). Impact of convolutional neural network and FastText embedding on text classification. Multim. Tools Applic. 82, 5569–5585. 10.1007/s11042-022-13459-x

[B43] Unni R. A. Sebastian L. Rajalakshmi S. Siby S. (2021). Detecting the presence of cyberbullying using machine learning. Int. J. Eng. Res. Techn. 9. 10.17577/IJERTCONV9IS13022

[B44] Wang K. Cui Y. Hu J. Zhang Y. Zhao W. Feng L. . (2021). Cyberbullying detection, based on the fasttext and word similarity schemes. ACM Trans. Asian Low-Resour. Lang. Inf. Process. 20, 1–15. 10.1145/3398191

[B45] Yao M. Chelmis C. Zois D. S. (2019). “Cyberbullying ends here: towards robust detection of cyberbullying in social media,” in The World Wide Web Conference (San Francisco, CA, USA: ACM), 3427–3433. 10.1145/3308558.3313462

[B46] Yuvaraj N. Chang V. Gobinathan B. Pinagapani A. Kannan S. Dhiman G. . (2021). Automatic detection of cyberbullying using multi-feature based artificial intelligence with deep decision tree classification. Comput. Electr. Eng. 92:107186. 10.1016/j.compeleceng.2021.107186

[B47] Ziems C. Vigfusson Y. Morstatter F. (2020). “Aggressive, repetitive, intentional, visible, and imbalanced: refining representations for cyberbullying classification,” in Proceedings of the International AAAI Conference on Web and Social Media, 808–819. 10.1609/icwsm.v14i1.7345

[B48] Zulqarnain M. Ghazali R. Hassim Y. M. M. Rehan M. (2020). A comparative review on deep learning models for text classification. Indones. J. Electr. Eng. Comput. Sci. 19:325. 10.11591/ijeecs.v19.i1.pp325-335

